# Antinociceptive and anxiolytic-like effects of a *neo*-clerodane diterpene from *Salvia semiatrata* aerial parts

**DOI:** 10.1080/13880209.2020.1784235

**Published:** 2020-07-07

**Authors:** Nancy Ortiz-Mendoza, Lizeth M. Zavala-Ocampo, Martha J. Martínez-Gordillo, María Eva González-Trujano, Francisco A. Basurto Peña, Iván J. Bazany-Rodríguez, José Alberto Rivera Chávez, Alejandro Dorazco-González, Eva Aguirre-Hernández

**Affiliations:** a Laboratorio de Productos Naturales, Departamento de Ecología y Recursos Naturales, Facultad de Ciencias, Universidad Nacional Autónoma de México, CDMX, México; b Herbario de la Facultad de Ciencias, Departamento de Biología Comparada, Facultad de Ciencias, Universidad Nacional Autónoma de México, CDMX, México; c Laboratorio de Neurofarmacología de Productos Naturales, Dirección de Investigaciones en Neurociencias, Instituto Nacional de Psiquiatría Ramón de la Fuente Muñiz, CDMX, México; d Jardín Botánico, Instituto de Biología, Universidad Nacional Autónoma de México, CDMX, México; e Instituto de Química, Universidad Nacional Autónoma de México, CDMX, México

**Keywords:** Anxiety, Lamiaceae, neoclerodanes terpenoids, nociception, salvias, traditional medicine

## Abstract

**Context:**

*Salvia semiatrata* Zucc. (Lamiaceae) is a species used as a tranquilizer and to relieve pain in folk medicine in Santiago Huauclilla, Oaxaca, Mexico.

**Objective:**

To evaluate the antinociceptive and anxiolytic-like effects of *S. semiatrata* extracts and identify a bioactive metabolite.

**Materials and methods:**

The extracts were obtained by maceration of *S. semiatrata* aerial parts using solvents in increasing polarity (hexane, ethyl acetate and methanol). A *neo-*clerodane diterpene was extracted from the ethyl acetate fraction using open column chromatography. Identification of this metabolite was performed by crystallography, ^1^H NMR, ^13^C NMR, ATR-IR, ECD, MS and elemental analysis. The antinociceptive activity was explored using the writhing and formalin tests. Whereas, the anxiolytic-like responses were analysed in the open-field, hole-board and plus-maze tests. All the treatments were administered using oral gavage in male CD1 mice and explored 30 min after administration of the individual extracts (300 mg/kg) or the compound **1** (0.1, 1 or 10 mg/kg).

**Results:**

All the extracts produced significant reduction in the nociceptive and anxiety-like behaviour compared to mice treated with the vehicle (0.5% tween 80 in saline solution). The spectroscopic analysis corroborated the presence of the *neo*-clerodane diterpene 7-keto-neoclerodan-3,13-dien-18,19:15,16-diolide (**1**), as partial responsible of the antinociceptive and anxiolytic-like effects, which produced a dose-dependent response in the writhing test with an ED_50_=4.15 mg/kg.

**Discussion and conclusions:** These results reinforce the medicinal properties of *S. semiatrata* in folk medicine, where participation of a *neo*-clerodane diterpene was evidenced in the inhibitory central nervous system activity of this species.

## Introduction

*Salvia* is the most diverse genus of the Lamiaceae family. In Mexico, it is represented by 306 species, of which 236 (77%) are endemic and most of them belong to the subgenus *Calosphace* (Martínez-Gordillo et al. 2017). From the past to the present time, sages have been used for their wide spectrum of medicinal properties in folk Mexican medicine. It is known that species, such as *Salvia coccinea* Juss. ex Murray, *Salvia cinnabarina* M. Martens & Galeotti, *Salvia fulgens* Cav., *Salvia microphylla* Kunth, *Salvia purpurea* Cav., *Salvia lavanduloides* Kunth, *Salvia hispanica* L., *Salvia rubiginosa* Benth., *Salvia urica* Epling and *Salvia elegans* Vahl are used to treat gastrointestinal alterations; for example, dysentery, diarrhoea, stomachache, inflammation and cramping (Domínguez-Vázquez and Castro-Ramírez 2002; Jenks and Seung-Chul 2013). While, *S. coccinea*, *Salvia divinorum* Epling & Játiva, *S. elegans*, *S. fulgens*, *S. lavanduloides*, *Salvia leucantha* Cav., *S. microphylla* and *Salvia polystachya* Ortega have been reported for their tranquilizing effects and their use to treat insomnia (Jenks and Seung-Chul 2013; Casselman et al. 2014).

Phytochemical studies in sages have reported the presence of monoterpenes, sesquiterpenes, diterpenes, triterpenes and phenolic compounds (Wu et al. 2012; Jenks and Seung-Chul 2013). However, it is worth noting the abundant presence of *neo*-clerodane diterpenes in American species (Rodríguez-Hahn et al. 1995; Esquivel 2008).

Preclinical studies of extracts and compounds (ursolic acid, β-sitosterol, cirsiliol and apigenin) from *Salvia* species have demonstrated several pharmacological effects at level of the central nervous system (CNS), such as anxiolytic, sedative, antidepressant and antinociceptive, among others (Imanshahidi and Hosseinzadeh 2006). Anti-inflammatory response has been included in the antinociceptive effects explored in *Salvia plebeia* R. Br. (Jung et al. 2009), *Salvia officinalis* L. (Rodrigues et al. 2012) and *Salvia circinata* Cav. (Moreno-Pérez et al. 2019). The antinociceptive effects of *S. officinalis* (Ghorbani and Esmaelizadeh 2017) and *S. circinata* (Moreno-Pérez et al. 2019) have been associated with the presence of diterpenes, triterpenes and flavonoids. In addition, the hydroalcoholic extract of *Salvia reuterana* Boiss. and the essential oil of *Salvia miltiorrhiza* Bunge produced sedative and anxiolytic-like effects (Rabbani et al. 2005; Liu et al. 2015), whereas antidepressant and anxiolytic-like activities were informed by *S. elegans* (Herrera-Ruiz et al. 2006).

*Salvia semiatrata* Zucc. is a perennial shrub, 1–2 m tall, small leaves and purple inflorescences. This species is known in Santiago Huaclilla, Oaxaca, with the common name of ‘mirto morado’ (purple myrtle). It is distributed in the dry areas of the state of Oaxaca and is used by residents, as a healing, anti-inflammatory, for earache, stomach-ache and for nervous disorders (Nambo 2015). From the medium polar extract of the *S. semiatrata* aerial parts, two *neo*-clerodane diterpenoids have been isolated and characterized, one of them was semiatrin and the other was 7α-hydroxy-*neo*-clerodan-3,13-dien-18,19:15,16-diolide (Esquivel et al. 1986; Soriano-García et al. 1987). From the root extract, the diterpenoids tilifolidione, horminone and 20-norinuroyleanol were also identified (Esquivel et al. 2005). Regarding biological activities, antifeedant effects of tilifolidione and horminone have been reported (Simmonds et al. 1996), as well as cytotoxic for tilifolidione (Esquivel et al. 2005) and antifungal and antibacterial for horminone (Ulubelen et al. 2001; Bufalo et al. 2016).

However, the pharmacological effects on the CNS of *S. semiatrata* or its metabolites are not found in the literature. The purpose of this research was to evaluate the antinociceptive and anxiolytic-like activities of several organic crude extracts of the *S. semiatrata* aerial parts and a bioactive metabolite (**1**) using several experimental models.

## Materials and methods

### Drugs and reagents

Ketorolac was purchased from SupraDOL^®^, Liomont (Mexico City, Mexico); Clonazepam was purchased from Gabaclotec^®^, Tecnofarma (Mexico City, Mexico); Diclofenac, acetic acid and 37% formalin were purchased from Merck (Mexico City, Mexico). Tween 80 and ceric sulphate were purchased from Sigma (St. Louis, MO). Hexane, ethyl acetate and methanol were purchased from Tecsiquim, S.A. de C.V. (Mexico City, Mexico). Drugs and methanol extract were dissolved in saline solution (0.9% NaCl, Sigma, St. Louis, MO). The hexane and ethyl acetate extracts, as well as the purified compound **1** were resuspended in Tween 80 at 0.5% in saline solution (vehicle). All treatments were freshly prepared and administered by oral gavage (p.o.). Nociceptive agents, 1% acetic acid and 1% formalin were diluted in saline solution and injected by intraperitoneal (i.p., 10 mL/kg) or intraplantar (20 µL/paw) route, respectively.

### Vegetal material

*S. semiatrata* aerial parts were collected in Santiago Huauclilla, Oaxaca, in July 2017. This region is located at the parallels 17°25′ and 17°34′ latitude north and meridians 96°56′ and 97°08′ longitude west, and at altitude between 1200 and 2700 m. A voucher specimen (no. 16362) was identified by Dra. Martha J. Martínez Gordillo and deposited in the IMSS Herbarium of CDMX, Mexico.

### Preparation of the extracts

**Figure 1.**
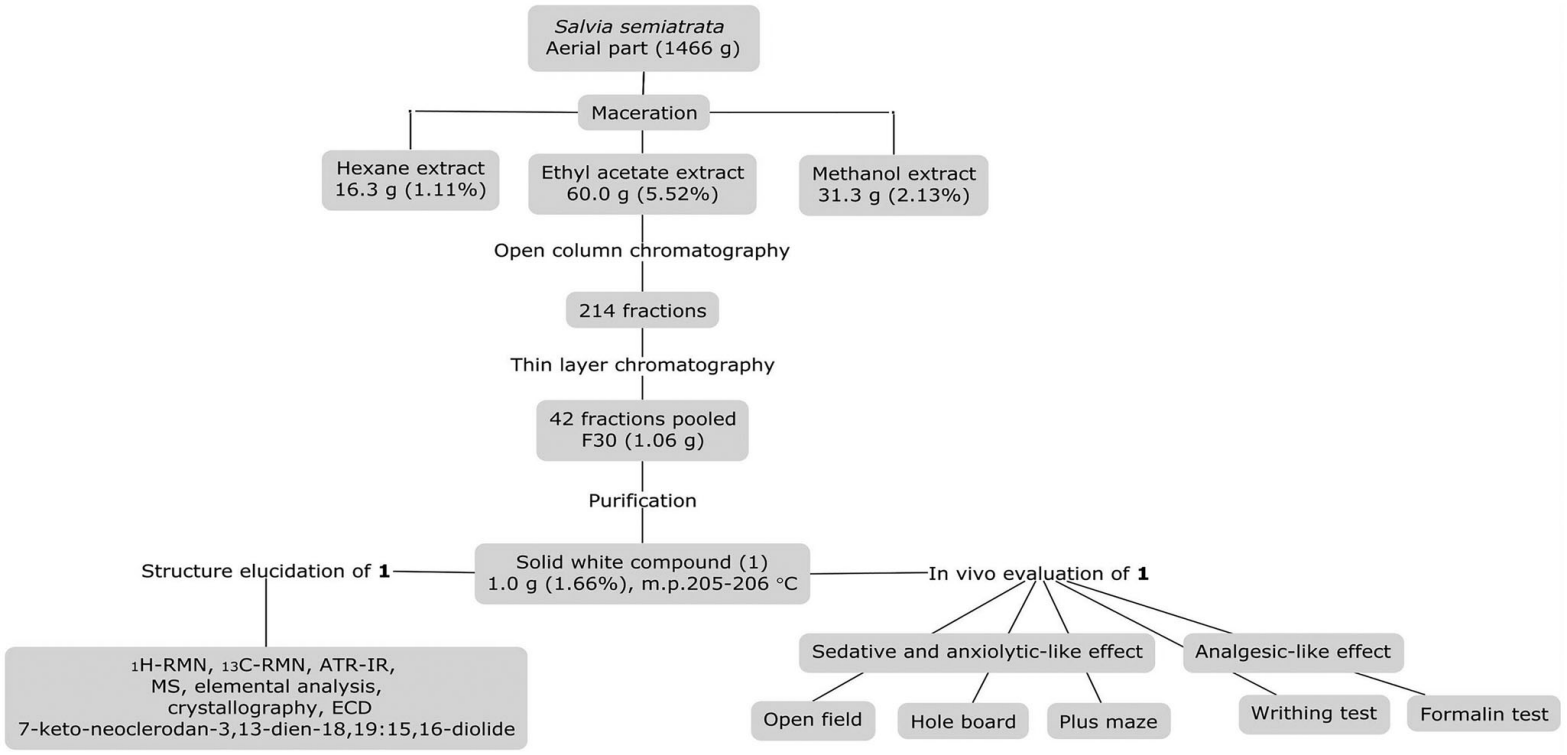
Diagram of the fractionation of the ethyl acetate extract of *S. semiatrata* aerial parts to isolate and identify the compound 7-keto-neoclerodan-3,13-dien-18,19: 15, 16-diolide (**1**).

Three organic extracts (hexane, ethyl acetate and methanol) of *S. semiatrata* aerial parts were obtained by a maceration process using 1466 g of the dry and ground plant material. For this, powder was kept in a container immersed in each solvent for 48 h at room temperature (22 °C). Each filtrate was concentrated under vacuum to eliminate solvents by evaporation in a rotary evaporator RII (Büchi Labortechnik AG, Flawil, Switzerland). A final yield of the extracts was obtained as follows: hexane (1.11%), ethyl acetate (5.52%) and methanol (2.13%) (Figure 1).

### Extraction and purification of a *neo*-clerodane diterpene (1)

The extraction of the active metabolite was carried out by fractionation of the ethyl acetate extract using conventional open-column chromatography packed with silica gel 60 GF_254_ (Macherey-Nagel, Düren, Germany) (Figure 1). The elution system consisted of 100% hexane, followed by hexane–ethyl acetate mixtures of increasing polarity until eluting with ethyl acetate (100%). Finally, it was eluted with 100% methanol. A total of 214 fractions of 100 mL each were collected, which were concentrated in a rotary evaporator for later analysis by thin layer chromatography. The plates were revealed with ceric sulphate to gather those with similar profile obtaining 42 subfractions.

Subfraction 30 eluted with the hexane–ethyl acetate mixture (3:7 v/v) was allowed to obtain a white crystalline precipitate with melting point (m.p.) 205–206 °C as measured in a Fisher-Johns apparatus (compound **1**). Compound **1** was crystallized with methanol–acetone and analysed by ATR-IR, ^13^C NMR, ^1^H NMR (Advance DPX400 Bruker Spectrometer, Billerica, MA) at 400 MHz, Circular Dichroism (Jasco J-1500, JASCO Inc., Easton, MD) and crystallography (Bruker Smart APEX II model diffractometer, Billerica, MA).

Compound **1** (1060 mg): white crystal, m.p. 205–206 °C.

IR (ATR) cm^−1^: 1767.44, 1739.03, 1701.44, 1663.29, 1636.19, 1165.25, 1023.63, 984.54, 877.76.

^1^H NMR (300 MHz, 25 °C, CDCl_3_): *δ* ^1^H NMR (300 MHz, CDCl_3_) *δ*: 6.87 (dd, *J* = 7.5, 2.0 Hz, 1H), 5.91 (t, *J* = 1.7 Hz, 1H), 4.79 (d, *J* = 1.9 Hz, 2H), 4.02 (d, *J* = 8.1 Hz, 1H), 3.93 (dd, *J* = 8.1, 2.3 Hz, 1H), 2.75 (d, *J* = 12.5 Hz, 1H), 2.63–2.47 (m, 2H), 2.44–2.23 (m, 5H), 1.91–1.64 (m, 3H), 1.37–1.20 (m, 1H), 1.02 (d, *J* = 6.7 Hz, 3H), 0.69 (s, 3H).

^13^C NMR (100 MHz, 25 °C, CDCl_3_): *δ* 208.51, 167.75, 166.92, 139.96, 136.28, 167.75, 168.52, 116.00, 72.93, 71.17, 51.49, 50.60, 48.05, 48.02, 43.61, 35.03, 27.45, 22.49, 20.47, 19.04, 8.00 (for the two signals of the –CH_3_ groups).

MS (FAB, *m*/*z*) 345, [MH]^+^. Anal. cal. for C_20_H_24_O_5_ (344.41): C, 69.75; H, 7.02. Found: C, 69.70, H, 7.06.

### Crystallographic investigations

Crystallographic data for **1** were collected on a Bruker APEX II CCD Diffractometer (Billerica, MA) at 150 K using Cu-Kα radiation (*k* = 1.54178 Å) from an Incoatec IµS source and Helios optic monochromator. Suitable crystals were coated with hydrocarbon oil (Parabar), picked up with a nylon loop, and mounted in the cold nitrogen stream of the diffractometer. The structure was solved using intrinsic phasing (SHELXT) (Sheldrick 2015) and refined by full-matrix least-squares on F2 using the shelXle GUI (Hübschle et al. 2011). The hydrogen atoms of the C–H bonds were placed in idealized positions. Crystallographic data were deposited at the Cambridge Crystallographic Data Center (CCDC) with the number 1953351.

### Computational details of ECD

3D model for compound **1** was built and geometry optimized using Spartan’10 with a PM3 semiempirical method. Conformational analysis was performed via Monte Carlo search protocol under the same force field. The conformers were checked for redundancy. All conformers within 10 kcal/mol were minimized using DFT at the B3LYP/DGDZVP level of theory employing Gaussian 09 (Gaussian, Inc., Wallingford, CT). The conformers were optimized, and the IR, vibrational analyses and thermochemical properties were obtained. The TD-SCF with default solvent model was used to perform the ECD calculations of the major conformers in MeOH solution, using a B3LYP-6-31 + G(d) force field. The calculated rotatory strength (*R*) in dipole velocity form (Rvel), and the excitation energy (nm) were simulated into an ECD curve using SpecDis software (Berlin, Germany), which uses the Harada–Nakanishi approximation (Equation (1)) (Bruhn et al. 2013). The spectra were generated with a *σ*/*γ* value of 0.28 eV and a UV correction of –5.0 nm. All calculations were performed on the HP Cluster Platform 3000SL ‘Miztli’, a parallel supercomputer with a Linux operating system, containing 25,312 cores and a total of 15,000 GB of RAM. The Harada–Nakanishi equation:

(1) $$\Delta \varepsilon (v)=\;\sum_{i=1}^{n}\Delta \varepsilon_{i}(v)=\sum_{i=1}^{n}=\;\frac{R_{i}}{{2.296\times 10}^{-39}\;\sqrt{\pi }}\frac{v_{i}}{\sigma }\;e[-{\left(\frac{v-v_{i}}{\sigma }\right)}^{2}]\;$$

### Pharmacological evaluation

#### Animals

Male CD1 mice (25–30 g of body weight) provided by the Bioterium of the Faculty of Sciences-UNAM were used in groups of six animals. Mice were placed in acrylic boxes with water and food *ad libitum* and kept at a controlled temperature of 22 ± 2 °C and with a 12 h light/dark cycle. The protocol was accepted on 4 June 2018 by the Research Ethics Committee for Animal Use of the Instituto Nacional de Psiquiatría Ramón de la Fuente Muñiz (COMBIOETICA-09.CEI-010-20170316). The experiments were carried out following national (NOM-062-ZOO-1999) and international ethical guidelines for the use of laboratory animals.

### Antinociceptive-like effects

Mice were divided into six groups to receive the following treatments by oral administration (p.o.): vehicle (0.5% Tween 80 in saline solution), the reference drug diclofenac or ketorolac (10 mg/kg, p.o.), the crude extract (hexane, ethyl acetate or methanol at 300 mg/kg, p.o.) or the *neo*-clerodane diterpene (0.1, 1 or 10 mg/kg, p.o.). Thirty minutes after the treatment, mice were independently subjected to the nociceptive agent to induce pain-like behaviour.

#### Writhing test

This test consisted of an i.p. administration of 1% acetic acid (0.1 mL/10 g body weight) to cause irritation in the peritoneal cavity, which in turn produced stereotyped behaviour in mice, where stretching of the hind limbs and abdominal contractions were characteristic. The number of stretches (writhes) was counted every 5 min for a total period of 30 min after injection of the nociceptive agent (Collier et al. 1968). The decrease in writhing behaviour was interpreted as an antinociceptive effect.

#### Formalin test

This test consisted of an intraplantar injection of 20 µL of 1% formalin in the right posterior paw of the mice to produce licking behaviour as painful reaction. Then, the time spent in licking the limb administered with the nociceptive agent was counted as a pain-like behaviour for 1 min every 5 min for a 30 min period. Two stages of licking behaviour, considered as painful response, were recorded in this test. The first was the early or neurogenic phase (0–10 min) and the second was the late or inflammatory phase (10–30 min) (Tjolsen et al. 1992). The decrease in licking behaviour in either phase was interpreted as an antinociceptive effect.

### Evaluation of the anxiolytic-like effects

Mice were divided into groups of six animals to receive one of the following treatments: vehicle (0.5% tween 80 in saline solution), the reference drug clonazepam (0.5 mg/kg, p.o.) and the organic extracts (hexane, ethyl acetate or methanol at 300 mg/kg, p.o.) or the *neo*-clerodane diterpene (0.1, 1 or 10 mg/kg, p.o.). Thirty minutes after the treatment, animals were consecutively tested in the anxiety models, such as open-field, hole-board, and finally in the plus-maze test.

#### Open field test

This test consisted of placing mice in an individual manner in the acrylic box divided into 12 squares (6 × 6 cm) to record the number of squares explored by each animal in a 2 min period (Prut and Belzung 2003). A significant decrease exploration was indicative of the sedative-like effects.

#### Hole-board test

This test consisted of an acrylic box with wooden floor with evenly distributed holes. Mice were placed on the board and the number of times the animals put their head into the holes in a period of three min was counted. The decreased exploration is indicative of the anxiolytic-like effects (Lister 1990).

### Plus-maze test

This device is made of wood and consists of an elevated cross with two open arms, 30 × 5 cm and two closed arms of 30 × 5×15 cm. These arms extended from a central platform of 5 × 5 are elevated 50 cm from the ground. Mice were individually placed in the junction of the four arms of the maze, facing an open arm. Then, the time that animals remained in the open arms for a 5 min period was counted (Lister 1987). The increased examination in open arms was indicative of the anxiolytic-like effects.

## Statistical analysis

Data from pharmacological evaluations are presented as the mean ± standard error of the mean (S.E.M.) of each treatment. Statistical analysis was carried out using one-way analysis of variance (ANOVA) followed by Dunnett’s *post hoc* test to compare treatments against the vehicle group. Data from the temporal course curve of writhing test were analysed by two-way ANOVA followed by Dunnett’s *post hoc* test. The analysis was done using GraphPad version 6 program (GraphPad Software, La Jolla, CA). A value of *p* < 0.05 was considered significant.

## Results

### Fractionation, purification and structural elucidation of a *neo*-clerodane diterpene

**Figure 2.**
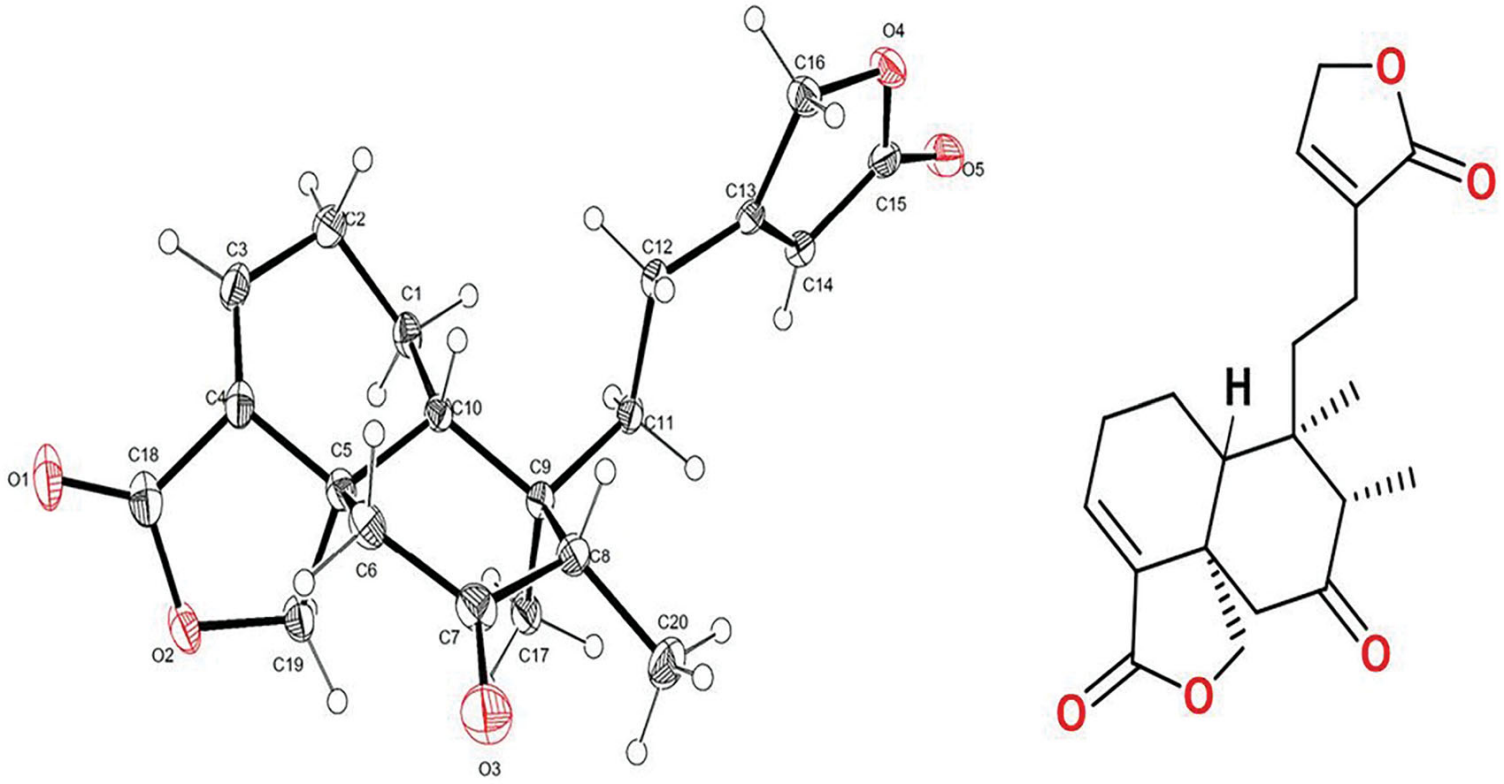
Perspective views of the molecular and chemical structure of the compound **1**. Ellipsoids are shown at the 50% probability level.

From the pharmacological evaluation of the extracts, it was obtained that the medium polar extract showed the highest yield, being 5- and 2-fold more abundant than the hexane and methanol, respectively (Figure 1). The chromatographic separation of the ethyl acetate extract lead to the isolation of the of the most abundant metabolite, identified as 7-keto-*neo*-clerodan-3,13-dien-18,19: 15,16-diolide (**1**), a *neo*-clerodane diterpene. This compound was purified for the first time from *S. semiatrata* as a crystalline form (Figure 2).

**Figure 3.**
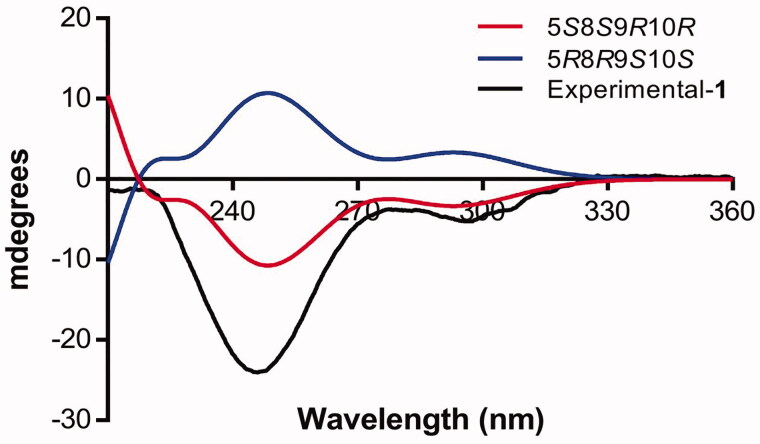
Experimental and calculated ECD spectra for 1-*5S8S9R10R* (red line) and 1-*5R8R9S10S* (blue line). A negative shift of 5 nm was required to match the spectra.

**Figure 4.**
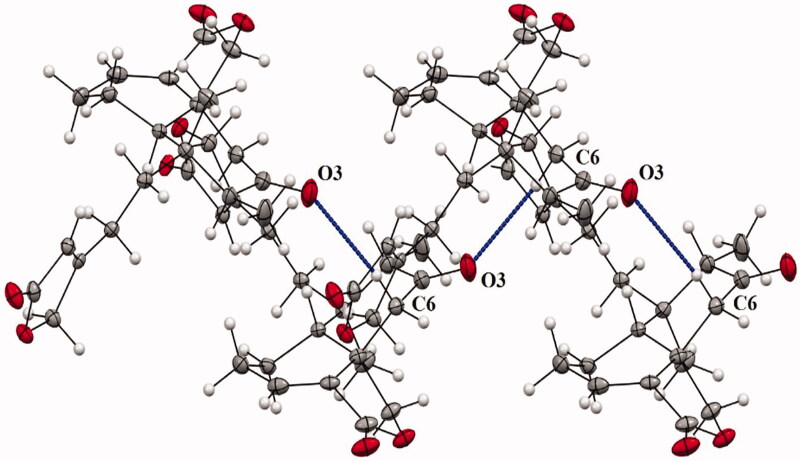
Stick model of a portion of the X-ray crystal of **1**. Dashed lines indicated inter-chain contacts, C (6)–H (6)…O (3) (from cyclohexanone ring).

The compound **1** was isolated and crystallized as white rectangles grown by slow solvent evaporation from a chloroform solution, m.p. (205–206 °C). The compound **1** was considered pure according to ^1^H NMR and ^13^C NMR spectra (Supplementary Figures 1–3). Spectroscopic data agreed to those previously reported for this compound isolated from *Baccharis* species (Asteraceae), such as *Baccharis trinervis* Pers. (Kuroyanagi et al. 1993), *Baccharis trimera* (Less.) DC. (Herz et al. 1977) and *Baccharis gaudichaudiana* DC. (Akaike et al. 2003). The ATR-IR spectrum (Supplementary Figure 4) showed two strong bands for α,β-unsaturated γ-lactone and one band for carbonyl group (1767, 1739 and 1701 cm^−1^). Here in, we reported for the first time full structural details of the *trans*-neoclerodane diterpene **1** by X-ray single-crystal analysis (Supplementary Table 1). Figure 2 shows a perspective view of the molecular structure of **1** where the compound crystallized in a monoclinic system with the P2_1_ space group. The cyclohexene and cyclohexanone rings are *trans*-fused and adopt twist boat and chair conformations, respectively. The γ lactone has an envelope conformation and the 13(16H) furanone ring is practically planar. The absolute stereochemistry at C-5, C-8, C-9 and C-10 was determined as S, S, R and R, respectively, based on the comparison of the calculated and experimental circular dichroism (ECD) spectrum (Figure 3), as well as the Flack parameter was 0.06. The molecular models for the most stable conformers (Supplementary Figure 5) of compound **1** were used to calculate the ECD spectrum. The experimental ECD spectrum for this compound showed two negative Cotton effects at 244 and 295 nm, which were in good agreement with those displayed for enantiomer *5S8S9R10R*. The methyl substituent at C-17 is axial and the methyl group C-20 occupies the more sterically favoured equatorial position. The crystal structure is stabilized by weak CH…O contacts forming infinite chains as it is shown in Figure 4. The C(6)…O(3) and H(6)…O(3) distances are 3.67(4) and 2.69(6) Å and the angle C(6)-H…O(3) is 168.6 (5)°.

### Antinociceptive effects of *S. semiatrata* crude extracts and the bioactive compound **1**

Much clinical pain occurs in deep muscle tissues and/or visceral organs, because of that, the acute and tonic pain models to specifically study muscle or visceral nociception are the writhing and formalin tests. To select the most active extract during the preliminary screening of the antinociceptive effect of the *S. semiatrata*, it was evaluated each in a single dose of 300 mg/kg, p.o. using both, writhing and formalin tests. In the writhing test, it was observed that all the extracts at 300 mg/kg, p.o. and the ketorolac (reference drug at 10 mg/kg, p.o.) produced significant reduction (*F*_4,25_ = 18.90, *p* < 0.001) in the nociceptive behaviour compared to mice treated with the vehicle (Table 1). The antinociceptive response was confirmed in the formalin test, where *S. semiatrata* extracts produced a significant reduction in the nociceptive response in both neurogenic (*F*_4,25_ = 11.57, *p* < 0.001) and inflammatory (*F*_4,25_ = 13.27, *p* < 0.001) phases of the test (Table 1).

**Table 1.** Antinociceptive and anxiolytic-like effects of the organic extracts from the *Salvia semiatrata* aerial parts.

		Antinociceptive effects			Anxiolytic-like effects		
Treatment/extracts	Dose (mg/kg)	No. of writhes	Licking time (s)		Open-field (counts/2 min)	Hole-board (counts/3 min)	Plus-maze (time in open-sided arms, s)
			1st phase	2nd phase			
Vehicle	–	105 ± 1.4	163 ± 32.3	368 ± 62.5	61 ± 2.1	23 ± 2.5	45 ± 6.5
Clonazepam	0.5	–	–	–	43 ± 1.8*	12 ± 1.8*	141 ± 9.3*
Ketorolac	10	20 ± 2.8*	–	–	–	–	–
Diclofenac	10	–	32 ± 7.6*	30 ± 10.8*	–	–	–
Hexane		51 ± 10.3*	80 ± 16.8*	190 ± 49.0*	47 ± 5.5	21 ± 1.5	136 ± 3.2*
Ethyl acetate		45 ± 10.0*	44 ± 3.6*	48 ± 19.6*	47 ± 7.0	17 ± 1.7	140 ± 7.9*
Methanol	300	73 ± 7.6*	82 ± 14.0*	92 ± 21.8*	56 ± 2.2	21 ± 2.7	87 ± 4.3*

Data represent mean ± S.E.M. of 6 animals. The asterisk indicates significant difference in comparison to the vehicle group. The antinociceptive response in writhing and formalin test (*p < 0.001). The anxiolytic-like effects: open-field (*p < 0.03), hole-board (*p < 0.001) and plus-maze test (*p < 0.0001) after ANOVA followed by Dunnett's post-hoc test.

**Figure 5.**
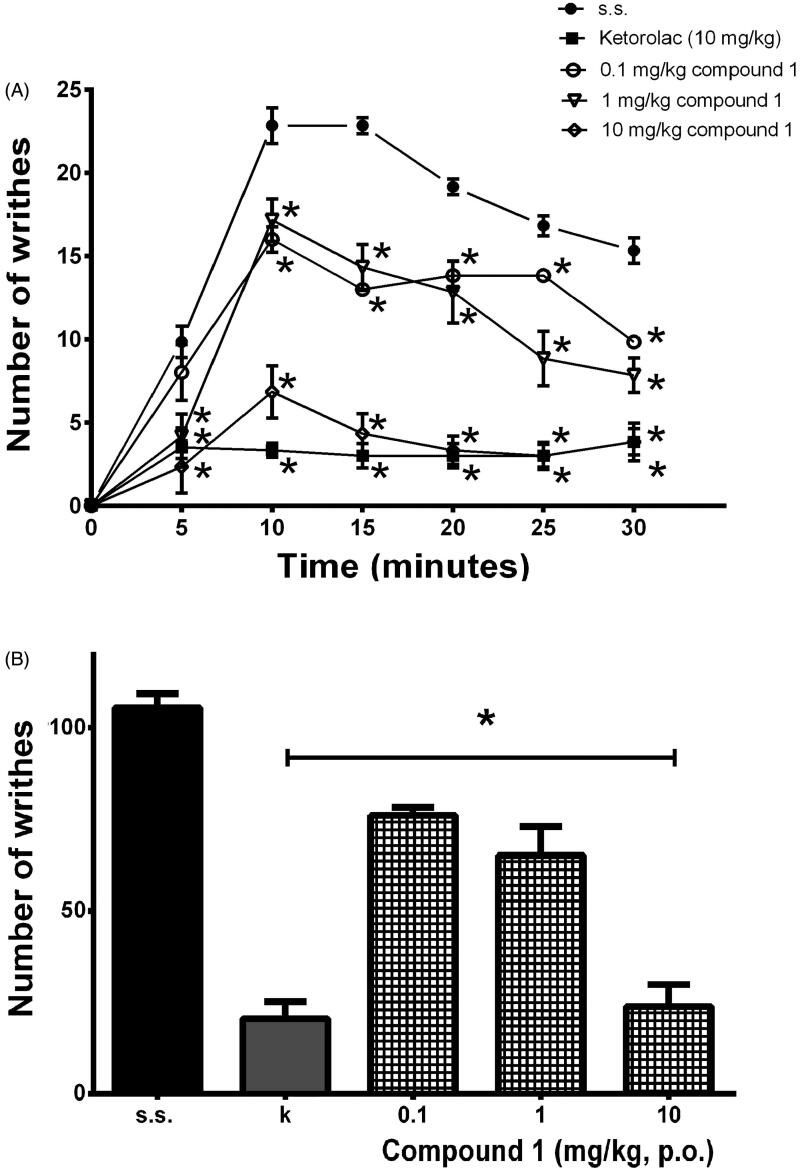
Antinociceptive-like effects of compound **1** and the reference drug (k, 10 mg/kg, p.o.) in the nociceptive response of the temporal course curve of the abdominal stretching (A, *p < 0.0001) and in the total stretching (B, *p < 0.001) induced by 1% acetic acid in mice. ANOVA followed by Dunnett’s *post hoc* test. An asterisk indicates significant difference in comparison to the vehicle group (s.s.).

Regarding compound **1**, it was purified for the first time from the *S. semiatrata* aerial parts as a crystalline form. According to the temporal course curves, the nociceptive response after administration of **1** was significantly reduced using doses of 0.1, 1 or 10 mg/kg, p.o. (*F*_4,25_ = 46.35, *p* < 0.001), it was observed from the first 5 or 10 min after administration of the algogenic agent (time: *F*_6,30_ = 129.3, *p* < 0.0001; treatment: *F*_4,20_ = 50.18, *p* < 0.0001; interaction: *F*_24,120_ = 19.23, *p* < 0.0001) (Figure 5(A)). The nociceptive behavioural response observed in the vehicle group was almost totally inhibited at the dosage of 10 mg/kg, p.o. of **1**. The effect was maintained throughout the entire period of the experiment resembling the antinociceptive response of the reference drug tested at the same dosage (Figure 5(A)). Antinociceptive response of **1** in the writhing test was observed in a dose-dependent manner producing an ED_50_ = 4.15 mg/kg (Figure 5(B)).

**Figure 6.**
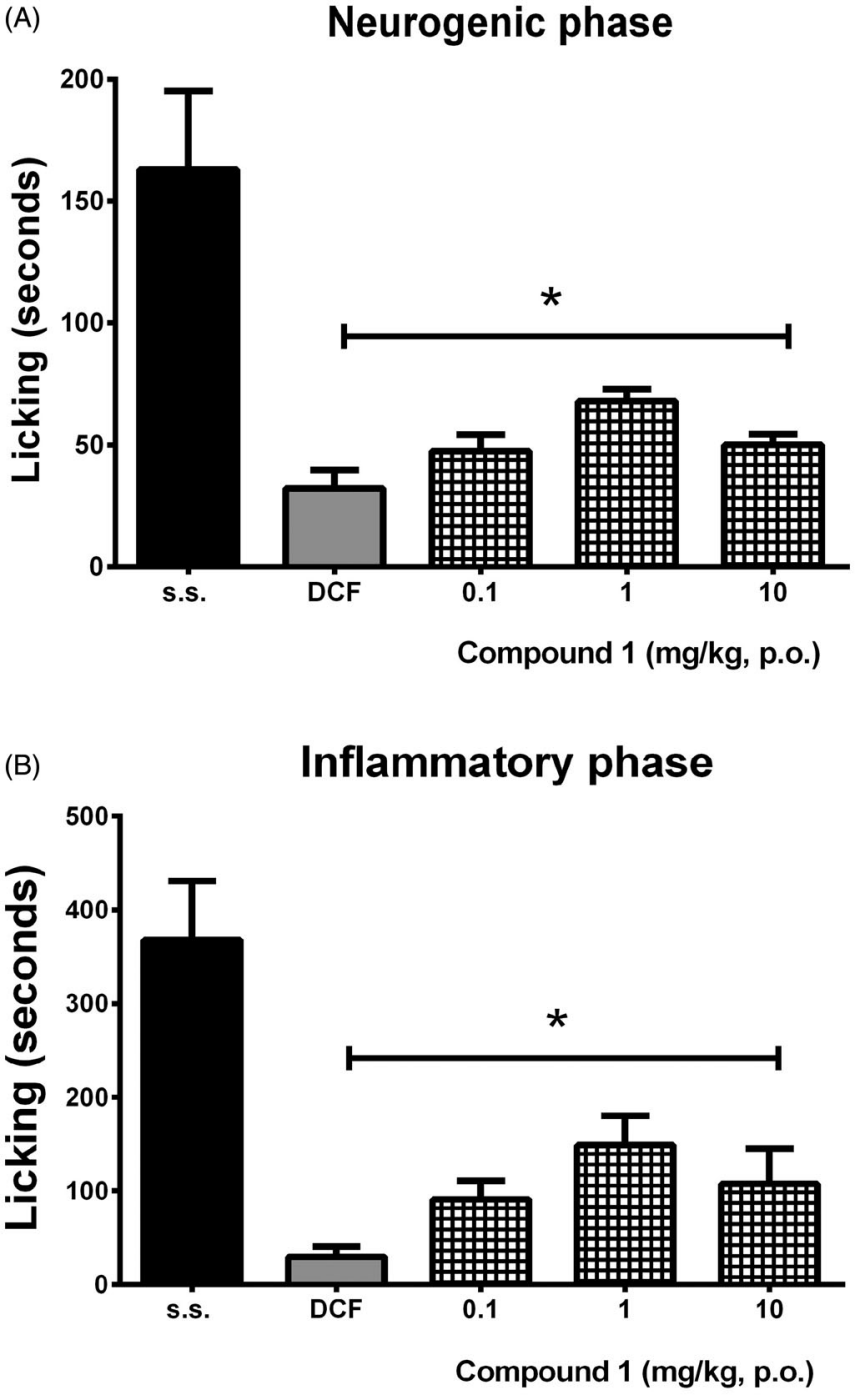
Antinociceptive-like effects of compound **1** and the reference drug (DCF, 10 mg/kg, p.o.) in the nociceptive response of time spent in licking in the neurogenic (A) and inflammatory (B) phases after intraplantar injection of 20 µL of 1% formalin in mice. ANOVA followed by Dunnett’s *post hoc* test. *p < 0.001 indicates significant difference in comparison to the vehicle group (s.s.).

Antinociceptive effects of **1** were also observed in the nociceptive response induced in the 1% formalin test. It was reduced in a significant manner at all doses tested resembling the effect of diclofenac, but this effect was not observed in a dose-dependent manner in both neurogenic (*F*_4,25_ = 11.57, *p* < 0.001) (Figure 6(A)) and inflammatory (*F*_4,25_ = 12.40, *p* < 0.001) (Figure 6(B)) phases like in the writhing test.

### Anxiolytic-like effects of *S. semiatrata* crude extracts and bioactive compound **1**

Anxiolytic-like effects of *S. semiatrata* were preliminary explored by administrating crude extracts (hexane, ethyl acetate or methanol) at a dosage of 300 mg/kg, p.o. The hexane and ethyl acetate extracts produced an equivalent significative anxiolytic-like response to that obtained with the reference drug clonazepam at 0.5 mg/kg, p.o. in the plus-maze test as compared to the vehicle group (*F*_4,25_ = 41.66, *p* < 0.0001) (Table 1). Tranquilizing response produced with all the extracts was not associated with the sedative-like effects, and it was not as intense like that observed with the reference drug in the open-field (*F*_4,25_ = 3.14, *p* < 0.03) and hole-board (*F*_4,25_ = 4.19, *p* < 0.001) tests (Table 1).

**Figure 7.**
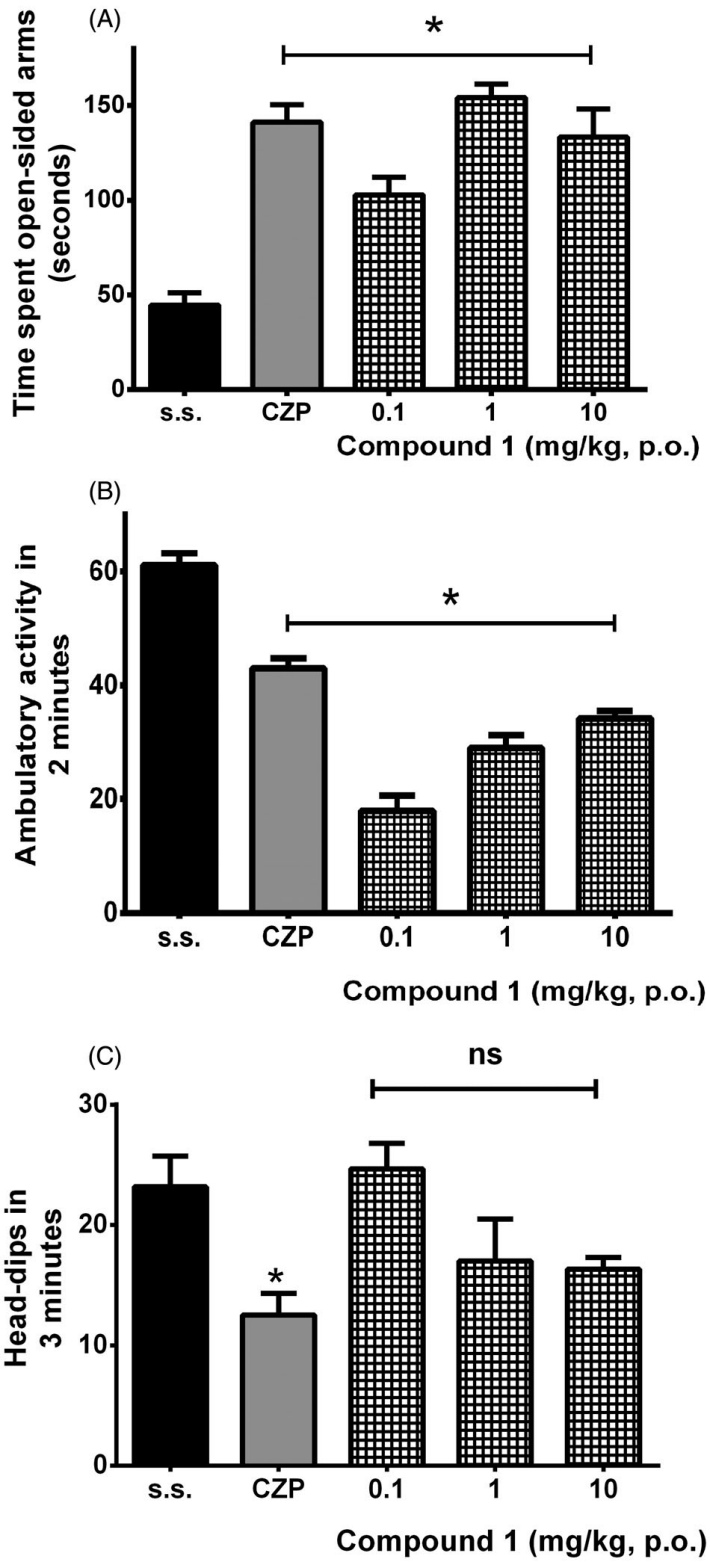
Significant anxiolytic-like effects of compound **1** and the reference drug (CZP, 0.5 mg/kg, p.o.) in comparison to the vehicle group (s.s.) were obtained in the plus-maze test (A, *p < 0.0001), open-field test (B, *p < 0.0001) and hole-board test (C, *p < 0.006) after ANOVA followed by Dunnett’s *post hoc* test. ns: non-significant differences.

In the case of compound **1**, it produced a significant increase in the open-sided arms examination resembling the reference drug clonazepam response in comparison to the vehicle group (*F*_4,25_ = 19.64, *p* < 0.0001) (Figure 7(A)). Unlike the extracts, compound **1** significantly modified ambulatory activity of mice in the open-field test (*F*_4,25_ = 62.91, *p* < 0.0001) (Figure 7(B)), but not in the dipping-like behaviour of the hole-board, where the highest dosage of **1** almost reached similar response to that observed with clonazepam (*F*_4,25_ = 4.72, *p* = 0.006) (Figure 7(C)). These results together suggest an increase in the tranquilizing effect when compound was administered.

## Discussion

The genus *Salvia* has been a source of medicinal plants with activity on the CNS (Imanshahidi and Hosseinzadeh 2006), regarding to the antinociceptive activity of different species of *Salvia.* It has been explored in several organic extracts. However, the medium polar extracts have demonstrated higher activity among them. For example, acetone extract showed greater reduction of the nociceptive response than methanol extract of *Salvia aegyptiaca* L. evaluated at doses from 250 to 2000 mg/kg, p.o. in the writhing and formalin tests in mice (Al-Yousuf et al. 2002). Hexane and chloroform extracts of *S. officinalis* leaves produced dose-dependent anti-inflammatory response by inhibiting croton oil-induced oedema in the mouse ear, where chloroform extract was the most active in comparison to a lower response obtained with methanol extract (Baricevic et al. 2001). In a similar manner, a chloroformic extract of *Salvia wiedemannii* Boiss. at 500 mg/kg, i.p. produced significant antinociceptive effect in the writhing and tail-flick tests in mice while, polar extracts like aqueous, ethanol or butanol were not effective (Ustun and Sezik 2011). Nevertheless, some *Salvia* species have been tested prepared only as a polar extract, for example, the ethanol of *S. plebeia* evaluated at doses of 50–200 mg/kg, p.o., which showed activity in abdominal pain and inflammation induced by carrageenan in mice (Jung et al. 2009). The antinociceptive effect in different types of nociception has been also reported for *S. divinorum*, partly due to the presence of salvinorins found in the medium polar extracts (Tlacomulco-Flores et al. 2020). All these results together suggest that medium polar constituents of *Salvia* species are mainly responsible of their antinociceptive effects and potential source of analgesic drugs.

Although there are several studies reporting the antinociceptive effects of different extracts and species of *Salvia*, there is not enough research on the main responsible active metabolites. In the case of *neo*-clerodane terpenes, there is also scarce information about its potential to relieve pain. An exception is *S. divinorum*, which is well known because of the presence of salvinorin A, a *neo*-clerodane terpene widely studied for its depressant activity on the CNS (Casselman et al. 2014). The dose-dependent antinociceptive effects of salvinorin A have been explored using the tail-flick and the hot plate tests (Tlacomulco-Flores et al. 2020), formalin (Guida et al. 2012) and writhing tests (McCurdy et al. 2006) in mice. In the present study, it should be noted that **1** tested at 10 mg/kg, p.o. produced a dose-dependent antinociceptive effect that remained until the end of the experiment (30 min) in the writhing test in mice (Figure 5(A)). Our data agree to that reported for tilifolidione (1–100 mg/kg, p.o.), a clerodane diterpene isolated from *Salvia tiliifolia* Vahl, which produced a dose-dependent antinociceptive effect in the writhing and formalin tests in mice (González-Chávez et al. 2018). Whereas, amarisolide (1, 5 and 10 mg/kg, i.p.), a *neo*-clerodane isolated from the aerial parts of *S. circinata*, produced significant but non-dose-dependent antinociceptive effects in the writhing test in mice (Moreno-Pérez et al. 2019). These data together reinforce the participation of *neo*-clerodane diterpenes as responsible for the depressant CNS activity of sage and their potential as alternative for pain treatment.

Anxiolytic and sedative-like activities in *Salvia* species and their bioactive metabolites have not been enough explored in preclinical or clinical studies. Nevertheless, anxiolytic-like effects for the essential oil of the *S. miltiorrhiza* aerial parts (100 and 200 mg/kg, p.o.) using the plus-maze test in rats (Liu et al. 2015) have been reported. While, this effect has also been observed in the hydroalcoholic extract (100 mg/kg, i.p.) of the *S. reuterana* aerial parts (Rabbani et al. 2005) and the flowers and leaves of *S. elegans* tested at 50 and 100 mg/kg, p.o. in mice, using both the plus-maze and open field tests, where ambulatory activity was not modified (Herrera Ruiz et al. 2006). An interesting case was that observed in *S. divinorum* leaves by an electroencephalography study in rats, which produced depressant activity and potentiation of the sodium pentobarbital-induced sleep. *S. divinorum* was prepared as hexane, ethyl acetate and methanol extracts and tested at doses of 10, 30 and 100 mg/kg, i.p. Its central effects were emphasized due to the presence of diterpenes such as salvinorins isolated in the medium polar extract (González-Trujano et al. 2016).

In general, anxiolytic drugs are intended to reduce anxiety or tension at doses which do not cause sedation or sleep. It is because oversedation, tremor, ataxia and confusion are the adverse effects much more common in patients consuming some anxiolytics drugs like benzodiazepines. Medicinal plants might produce its anxiolytic like activity without sedative effects at certain doses. In our study, to explore ambulatory and exploratory activities in mice at several doses was very important in order to identify if anxiolytic-like response is associated to sedative effects and at what range of doses it will be produced. Literature has reported compounds from different chemical nature, such as non-polar and polar, as responsible for the depressant-like activity of these medicinal plants, but diterpenoids have been the most diverse metabolites characterized in *Salvia* genus. According to their structure, these are classified into abietanes, labdanes, clerodanes, pimaranes and icetexanes. Biological activities, such as antimicrobial, antibacterial and cytotoxic, have been reported from these diterpenes (Wu et al. 2012). However, there are few studies which explore their effects on the CNS, specifically for *neo*-clerodanes diterpenes (Moreno-Pérez et al. 2019). The most studied case is salvinorin A, isolated from the leaves of *S. divinorum*, which has been reported for its therapeutic effects on Alzheimer’s or bipolar disorders (Casselman et al. 2014), as well as sedative (Fantegrossi et al. 2005), anxiolytic (Braida et al. 2009) and antinociceptive (McCurdy et al. 2006; Guida et al. 2012) activities. Salvinorin A is known as an agonist of the kappa opioid receptors, but it also possesses a partial affinity for CB1 cannabinoid receptors (Braida et al. 2009). It shows similar chemical structure than compound **1** and it is known to produce anxiolytic-like effects at doses of 0.1, 1.0, 40, 80 or 160 μg/kg, s.c. in the plus-maze test, without effects on the ambulatory activity of mice tested at 0.01, 0.1 and 1 μg/kg, s.c., probably because of the use of a lower dosage. We have not explored mechanism of action in the present study. However, to know the doses in which an anxiolytic drug produces its effects without sedation is very important since both sedative and anxiolytic drugs produce CNS inhibitory effects. Activation of the kappa opioid receptor relieves pain, but it is also associated to dysphoria (feeling of discomfort or aversion) and sedation (McDonald and Lambert 2005). Whereas, it has been reported that a mild activation of CB1 receptors in the prefrontal cortex and ventral hippocampus attenuates anxiety (Rubino et al. 2008). In the future, it will be interesting to explore about the mechanisms of action of compound **1** to produce its CNS depressant effects.

In conclusion, the present study gives evidence of the antinociceptive and anxiolytic-like effects of *S. semiatrata*, in part due to the presence of the diterpene *neo*-clerodane 7-keto-neoclerodan-3,13-dien-18,19: 15,16-diolide obtained for the first time as a crystal, reinforcing the medicinal properties of this species as a tranquilizer and for pain relief.

## Supplementary Material

Supplemental_material.docx

## Geolocation information

Laboratorio de Productos Naturales, Departamento de Ecología y Recursos Naturales. Facultad de Ciencias, Universidad Nacional Autónoma de México, Ciudad Universitaria Coyoacán, 04510, CDMX, México (19.324071, −99.180024).
