# Supplementary material for: Antinociceptive and anxiolytic-like effects of a neo-clerodane diterpene from Salvia semiatrata aerial parts
Source: Pharm Biol. 2020 Jul 7;58(1):620–9. doi: 10.1080/13880209.2020.1784235 (PMC7471977; doi:10.1080/13880209.2020.1784235)
Supplement: Supplemental_material.docx [file IPHB_A_1784235_SM3315.docx]

**Supplemental Material**

**Antinociceptive and anxiolytic-like effects of a *neo*-clerodane diterpene from *Salvia semiatrata* aerial parts**

Table 1. Crystal data and structure refinement for compound **1**

Empirical formula C_20_H_24_O_5_

Formula weight 344.39

Temperature 150(2) K

Wavelength 1.54178 Å

Crystal system Monoclinic

Space group P2**_1_**

Unit cell dimensions a = 10.6246(2) Å α= 90°.

b = 8.14190(10) Å β= 116.3760(10) °.

c = 11.0592(2) Å γ = 90°.

Volume 857.08(3) Å^3^

Z 2

Density (calculated) 1.334 Mg/m^3^

Absorption coefficient 0.778 mm^-1^

F (000) 368

Crystal size 0.400 x 0.180 x 0.120 mm^3^

Theta range for data collection 4.462 to 68.192°.

Index ranges -12<=h<=11, -9<=k<=9, -13<=l<=13

Reflections collected 13076

Independent reflections 3130 [R(int) = 0.0301]

Completeness to theta 67.679° 100.0 %

Absorption correction Semi-empirical from equivalents

Max. and min. transmission 0.7531 and 0.6569

Refinement method Full-matrix least-squares on F^2^

Data / restraints / parameters 3130 / 1 / 228

Goodness-of-fit on F^2^ 1.099

Final R indices [I>2sigma(I)] R1 = 0.0314, wR2 = 0.0715

R indices (all data) R1 = 0.0346, wR2 = 0.0735

Absolute structure parameter 0.06(7)

Largest diff. peak and hole 0.191 and -0.159 e.Å^-3^


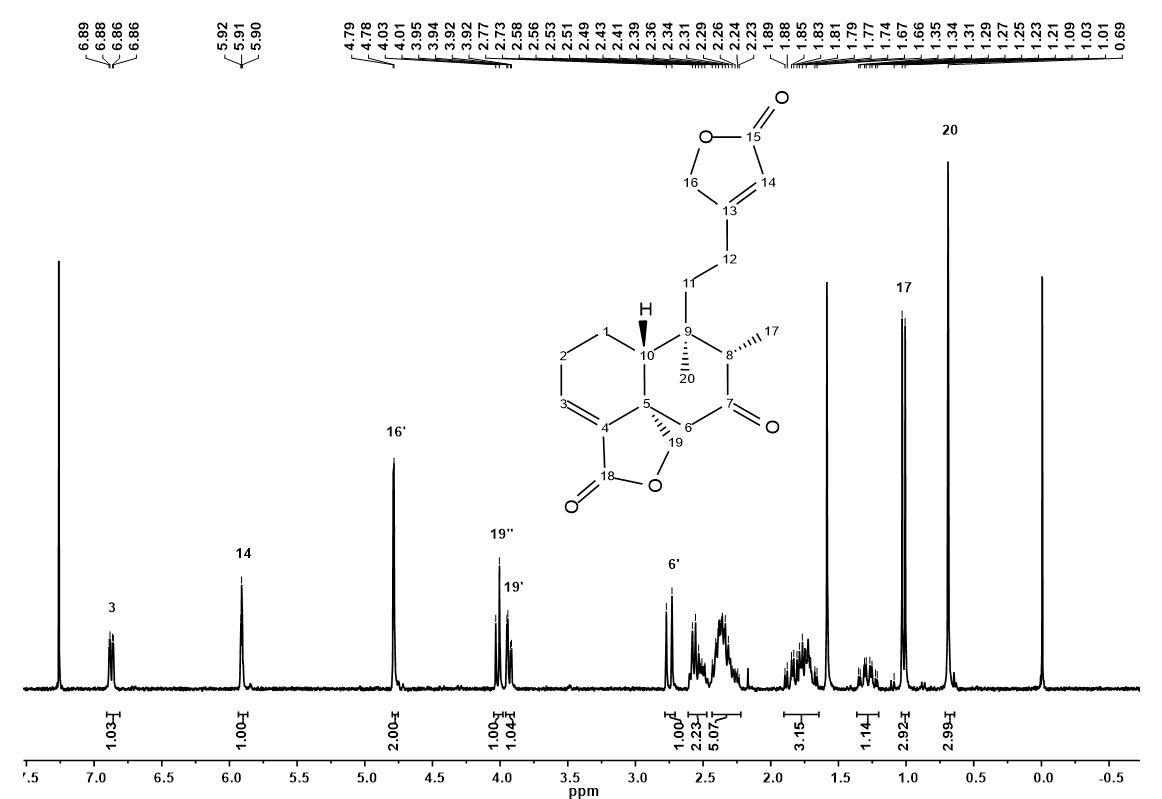


Figure 1. ^1^H-NMR (300 MHz) spectrum of **1** in CDCl_3_


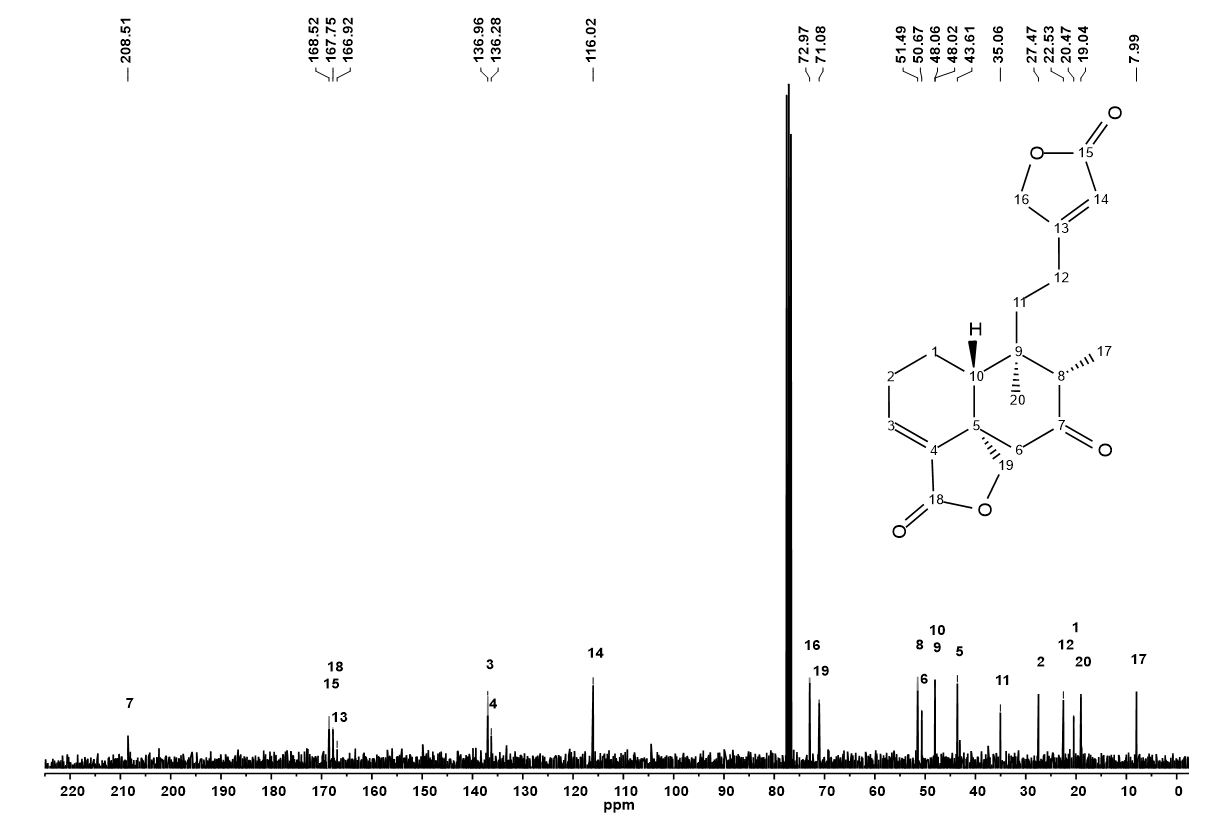


Figure 2. ^13^C-NMR (125 MHz) spectrum of **1** in CDCl_3_


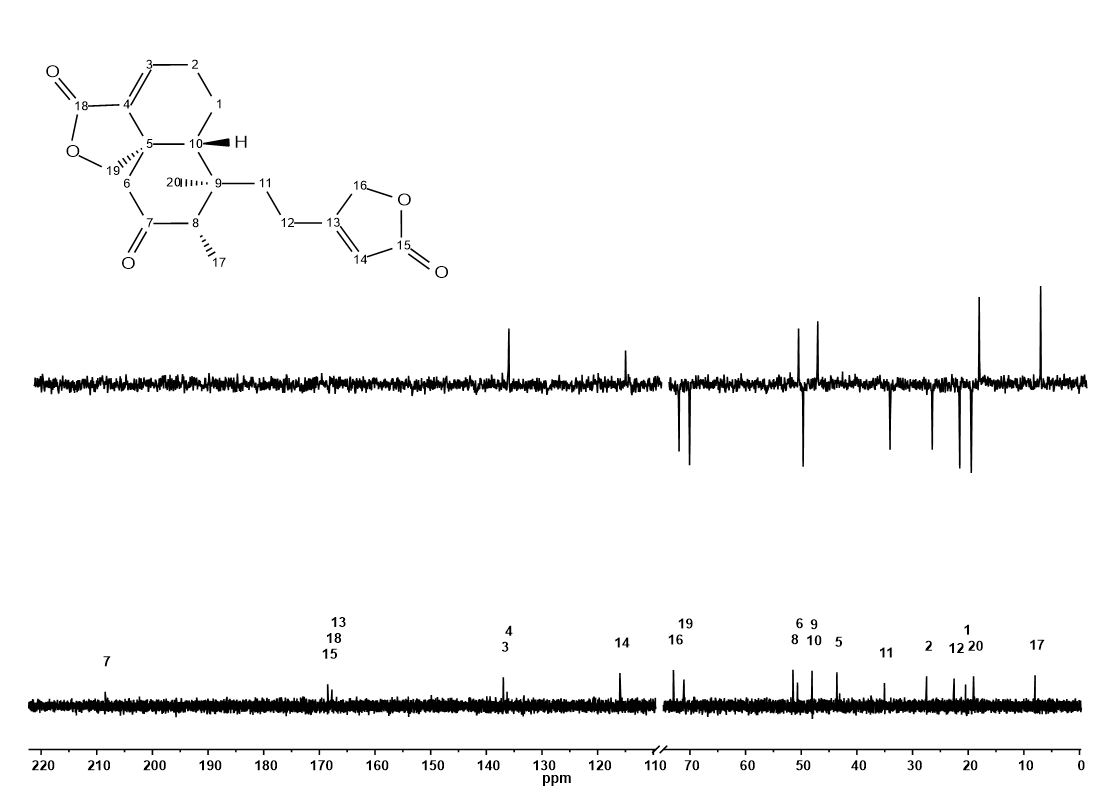


Figure 3. DEPT spectrum of **1** in CHCl_3_


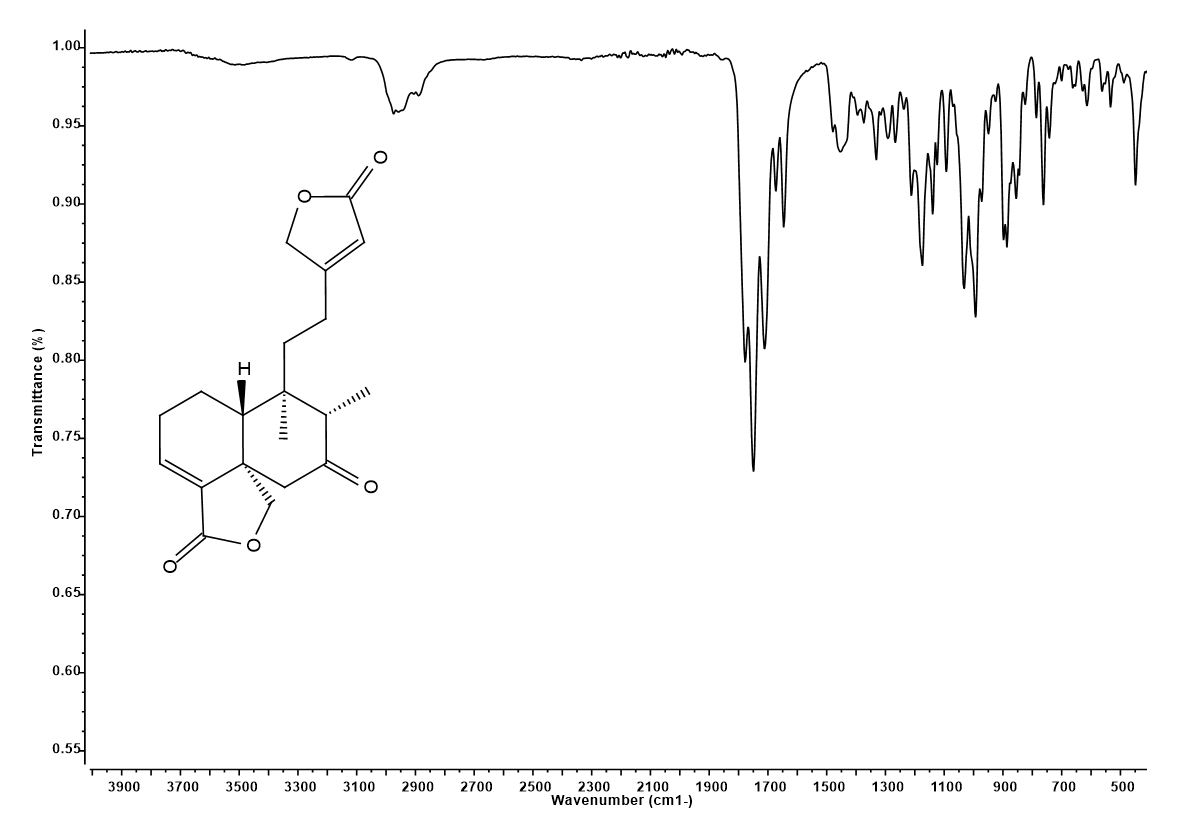


Figure 4. IR-ATR spectrum of **1**


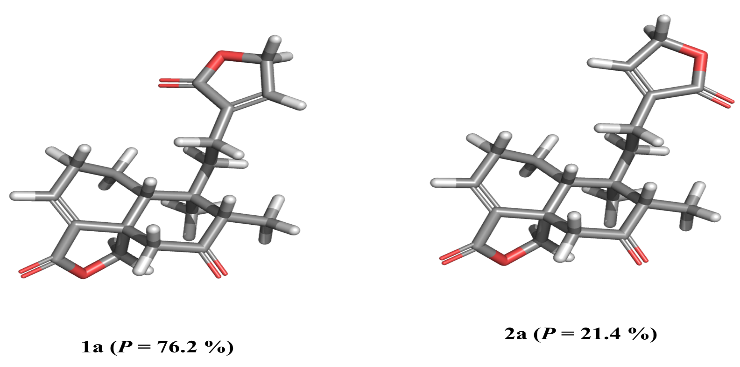


Figure 5. The most relevant conformers for compound **1** accounting for 97.6% of the conformational population, according with Boltzman distribution.
